# Supplementary material for: Scorpion Venom Antimicrobial Peptide Derivative BmKn2-T5 Inhibits Enterovirus 71 in the Early Stages of the Viral Life Cycle In Vitro
Source: Biomolecules. 2024 May 1;14(5):545. doi: 10.3390/biom14050545 (PMC11117539; doi:10.3390/biom14050545)
Supplement: Supplementary file 1 [file biomolecules-14-00545-s001.zip › biomolecules-2972149-supplementary.pdf]

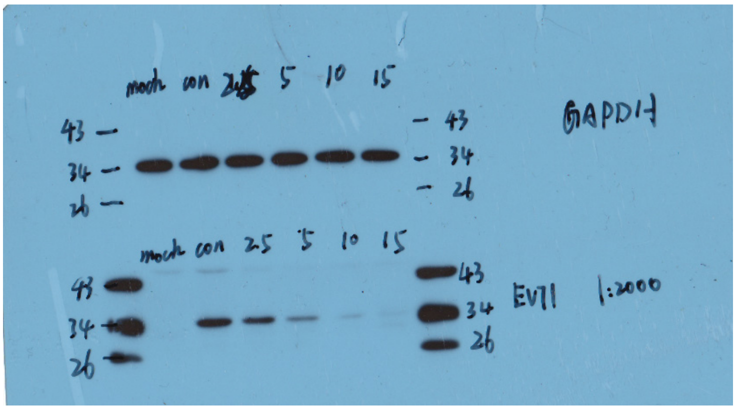

Figure S1. Dose-dependent inhibitory effect of BmKn2-T5 on EV71 analyzed using Western blotting (Original image).

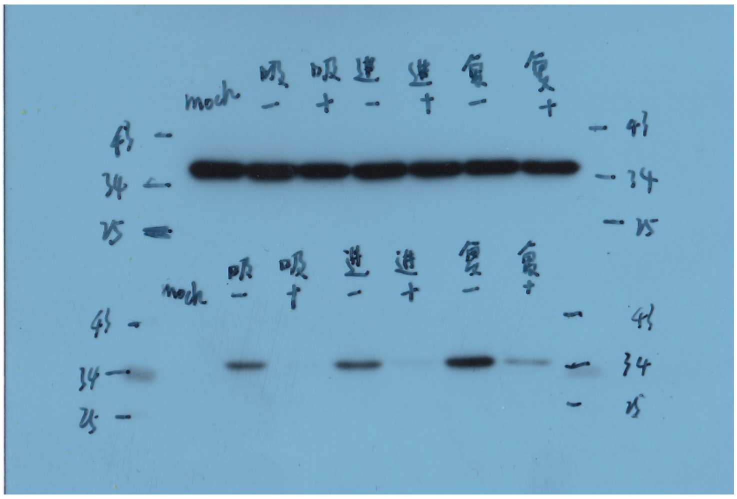

Figure S2. EV71 attachment, entry, and replication in RD cells detected using Western blotting (Original image).
